# Supplementary figures and images for: MRE11 Is Crucial for Malaria Parasite Transmission and Its Absence Affects Expression of Interconnected Networks of Key Genes Essential for Life
Source: Cells. 2020 Dec 3;9(12):2590. doi: 10.3390/cells9122590 (PMC7761864; doi:10.3390/cells9122590)

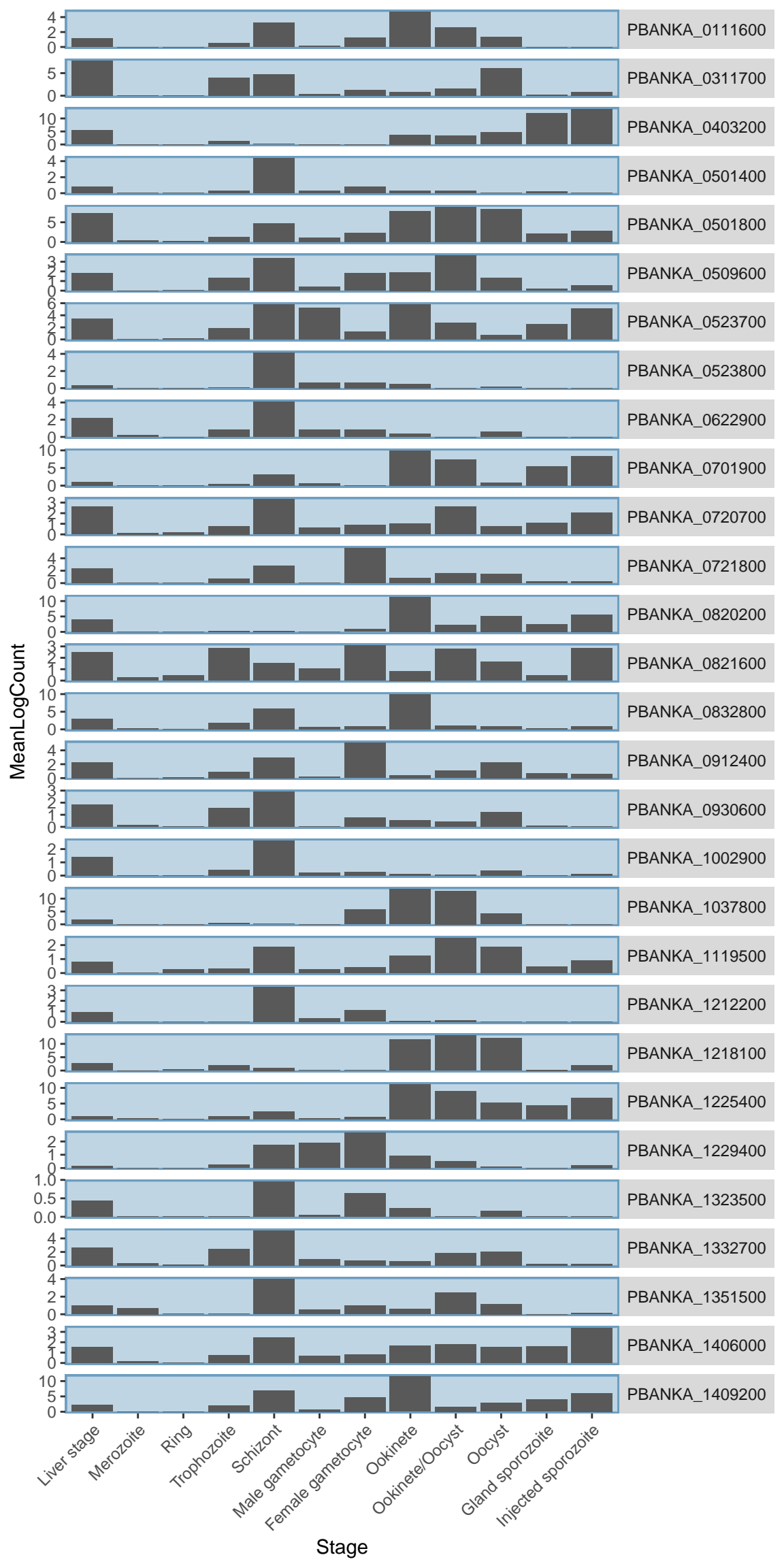

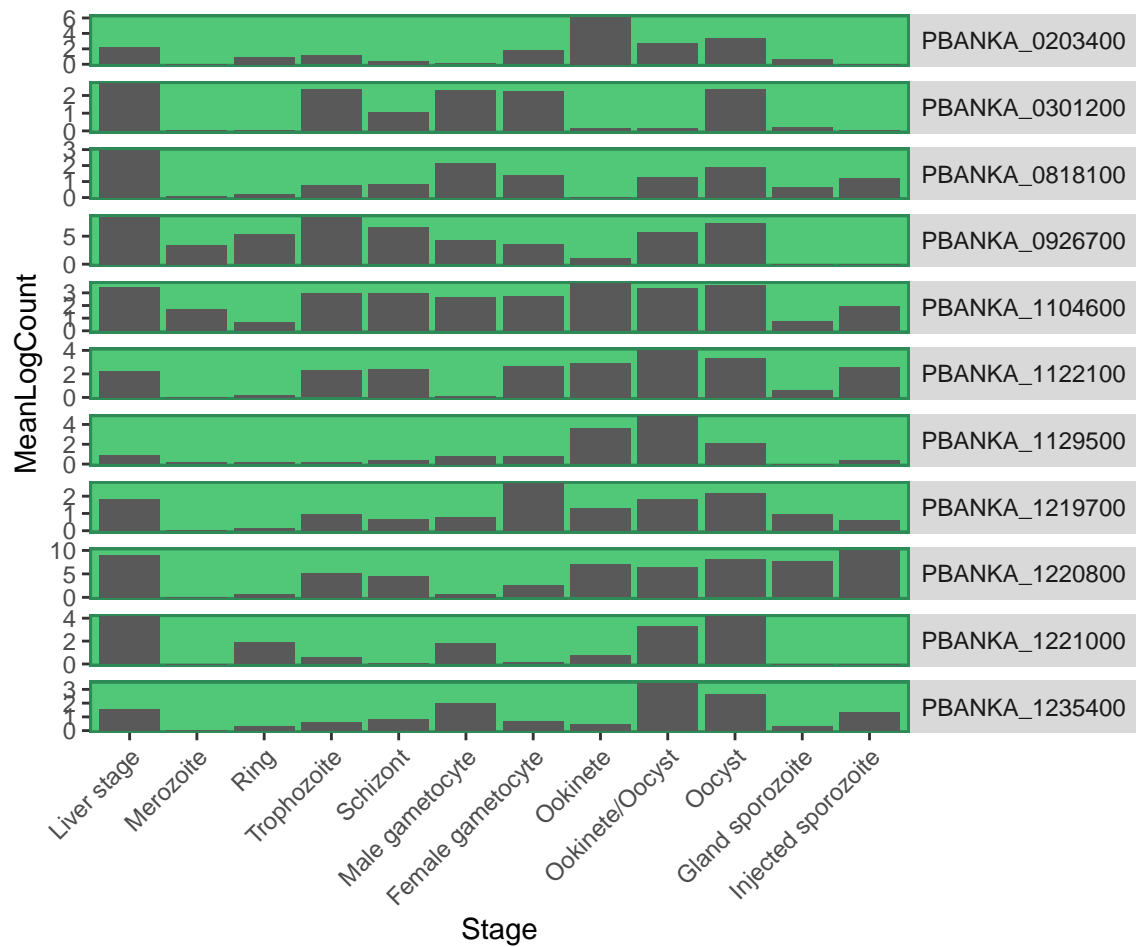

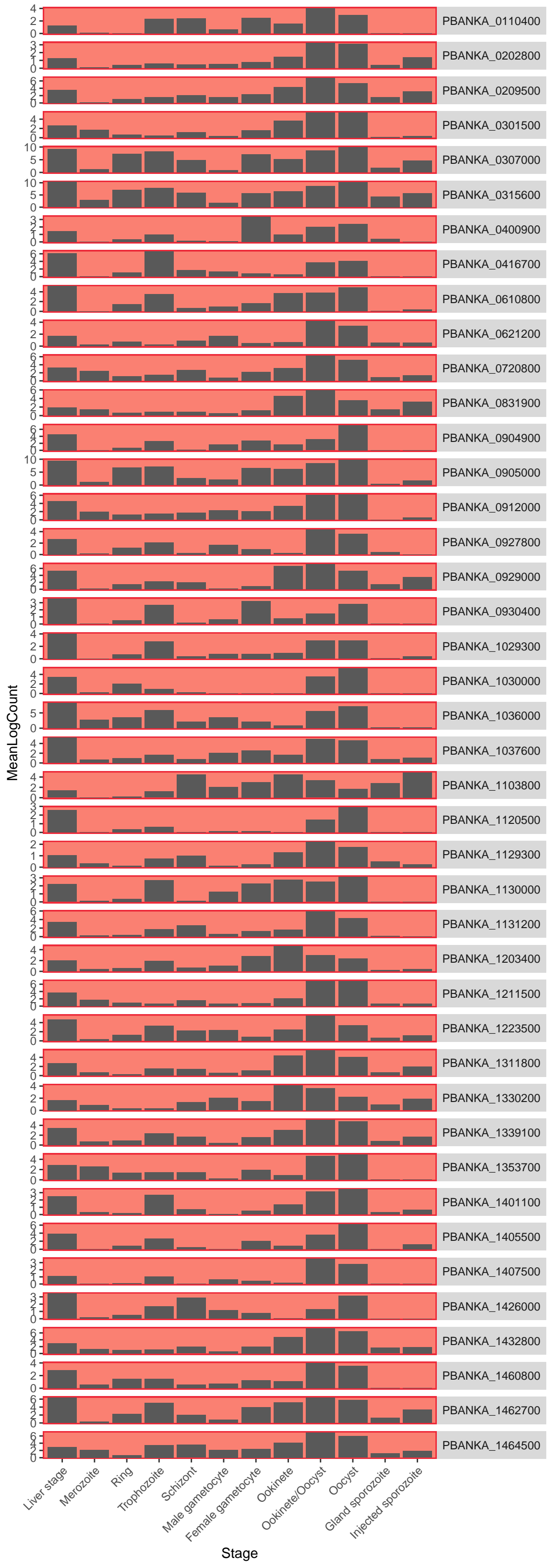

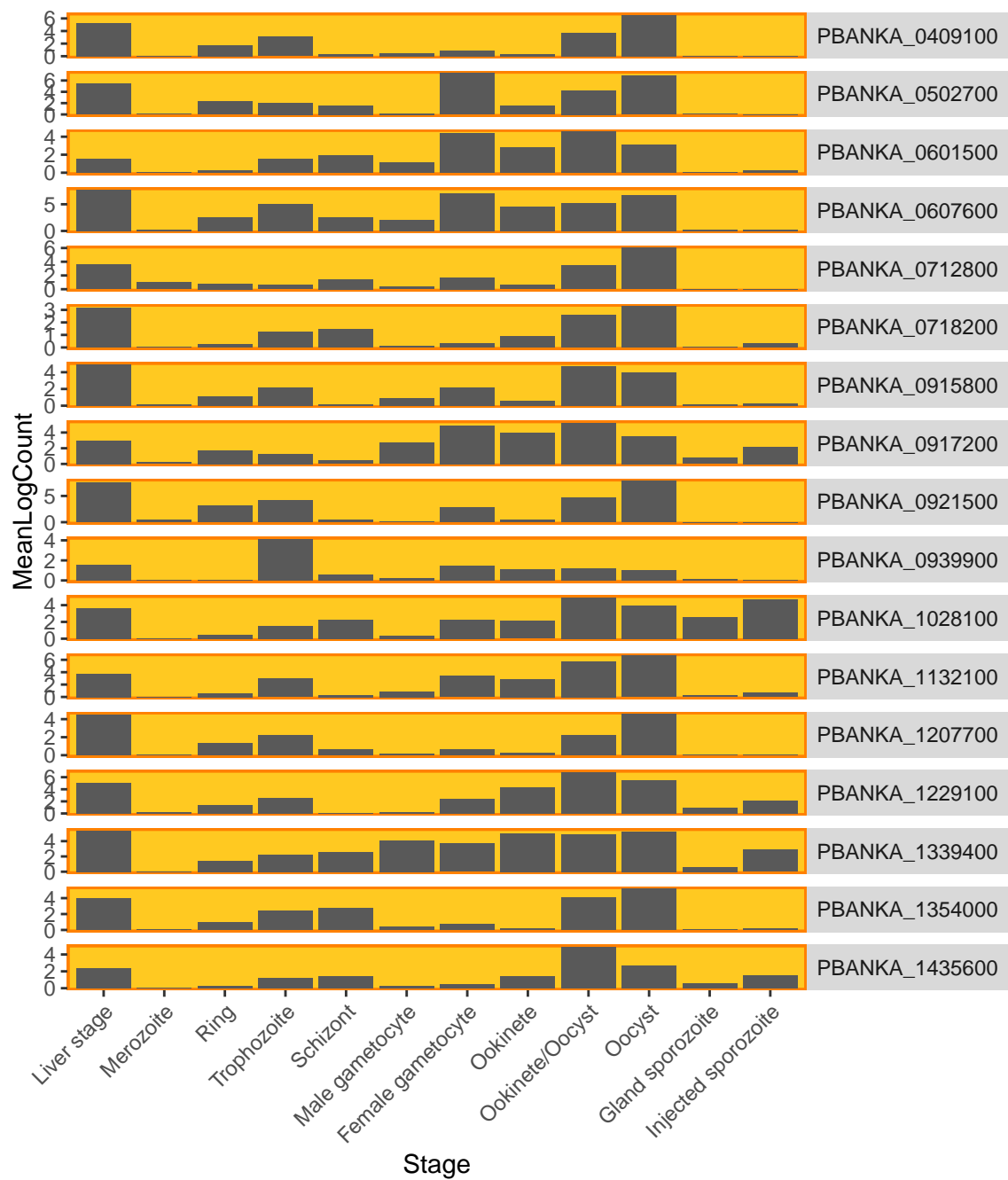

Supplement: Supplementary file 1 [file cells-09-02590-s001.zip › Data file S1.pdf]
